# Association of gut microbiota with lactose intolerance and coeliac disease: a two-sample Mendelian randomization study

**DOI:** 10.3389/fnut.2024.1395801

**Published:** 2024-08-06

**Authors:** Zongze Han, Ying Ran, Jiwen Li, Xue Zhang, Hui Yang, Jiangpeng Liu, Shijing Dong, Hao Jia, Zhen Yang, Yanni Li, Liping Guo, Simin Zhou, Suriguge Bao, Wei Yuan, Bangmao Wang, Lu Zhou

**Affiliations:** Department of Gastroenterology and Hepatology, General Hospital, Tianjin Medical University, Tianjin, China

**Keywords:** lactose intolerance, coeliac disease, gut microbiota, Mendelian randomization, genome-wide association study

## Abstract

**Background and objectives:**

Lactose intolerance and coeliac disease are common clinical nutrient malabsorption disorders, with an unclear pathogenesis and limited therapeutic options. It is widely believed that the gut microbiota plays an important role in many digestive disorders, but its role in lactose intolerance and coeliac disease is not yet clear. This study aimed to investigate the correlation between gut microbiota and lactose intolerance and coeliac disease.

**Materials and methods:**

This study utilized the genome-wide association study database to investigate the association between gut microbiota and lactose intolerance and coeliac disease using Mendelian randomization (MR). The robustness of our findings was confirmed through subsequent analyses including Cochrane’s *Q* statistic, MR-Egger Intercept Regression, MR-PRESSO Global Test and Leave-one-out methods.

**Results:**

By employing the inverse variance weighted method, we identified that family *Veillonellaceae*, genus *Oxalobacter* and *Senegalimassilia* were protective against lactose intolerance, whereas genus *Anaerotruncus*, *Eubacterium rectale group* and *Ruminococcus2* were found to be risk factors for lactose intolerance. Regarding coeliac disease, class *Bacilli* and *Gammaproteobacteria*, family *FamilyXIII* and *Veillonellaceae*, genus *Eisenbergiella*, *Lachnoclostridium*, *RuminococcaceaeUCG014* and *Ruminococcus2* were identified as protective factors, while class *Betaproteobacteria*, genus *Eubacterium xylanophilum group* and *Blautia* were risk factors. Furthermore, reverse the MR analysis did not reveal any evidence of a causal relationship between lactose intolerance or coeliac disease and the bacteria identified in our study.

**Conclusion:**

This study provides novel insights into exploring the role of gut microbiota in lactose intolerance and coeliac disease; however, further experiments investigations are required to elucidate the specific underlying mechanisms.

## 1 Introduction

Over the past two decades, there has been a significant increase in the number of cases of malabsorption disorders. In addition to intestinal symptoms such as bloating, abdominal pain or diarrhea, malabsorption disorders can also be complicated by fatigue, headache, depression and other extra-intestinal manifestations, leading to a decline in patients’ quality of life (1). Lactose intolerance and coeliac disease are common clinical nutrient malabsorption disorders, with an unclear underlying mechanism and limited therapeutic options.

Lactose intolerance is a disorder characterized by impaired lactose digestion and absorption due to reduced or absent lactase activity in the small intestinal mucosa, leading to a range of clinical symptoms including abdominal discomfort, bloating or gas, cramping, and diarrhea (2). It has been that up to 70% of the world’s population suffers from primary lactase deficiency (3). In addition to gastrointestinal symptoms, patients with lactose intolerance may experience parenteral symptoms, such as headache, joint and/or muscle pain, osteoporosis, eczema, oral ulcers, heart palpitations skin lesions and anxiety. These symptoms can have a negative impact on both quality of life and nutritional status (4, 5). Current management strategies for lactose intolerance are limited to reducing the intake of lactose-rich foods and utilizing lactose replacement medications (6). However, the clinical efficacy of lactase supplementation remains suboptimal (7).

Coeliac disease is a permanent T-cell-mediated enteropathy that primarily affecting the small intestine. It arises from genetic susceptibility combined with gluten ingestion (the major protein component in wheat, rye, and barley) in genetically susceptible individuals. The prevalence rate in the general population is approximately 1%. Individuals with a family history of coeliac disease face a lifetime risk ranging from 10 to 15% for developing this condition (8). Classic coeliac disease predominantly occurs in children under 5 years old and presents with chronic diarrhea, loss of appetite, weight loss, bloating, muscle wasting, and mood changes. Non-classical coeliac disease is commonly characterized by non-specific intestinal complaints such as recurrent abdominal pain, bloating, diarrhea, or constipation, as well as extraintestinal manifestations including persistent iron deficiency, chronic fatigue, dermatitis herpetiformis, or hypergammaglobulinemia (8, 9). In addition, the disease is associated with an elevated risk of psychiatric comorbidities, non-Hodgkin’s lymphoma, and intestinal adenocarcinoma (10). Lifelong adherence to a strict gluten-free diet is the only known effective treatment, creating an urgent need for more effective treatments (11).

Evidence has demonstrated a close association between gut microbiota and lactose intolerance as well as coeliac disease. The clinical manifestations of lactose intolerance are influenced by small intestinal bacterial overgrowth and the composition of the gut microbiome (12). Furthermore, studies have shown that gut microbiota is involved in lactose metabolism (13). Unabsorbed lactose alters intestinal osmotic pressure and the growth of gut microbiota. Gut microbiota, in turn, promotes the fermentation of unabsorbed lactose, leading to the production of hydrogen, methane and other gasses, that cause abdominal distension and pain (14). *Bifidobacterium* or other lactose-fermenting bacteria were reported to affect the production of lactose in the gut (12, 15). Gois et al. (13) analyzed data of intestinal disease, genetics, gut microbiome and diet from 959 Dutch participants, and observed an increase of *Bifidobacterium* abundance in lactose intolerance individuals compared with non-lactose-intolerance individuals. The abundance of adult gut bifidobacteria depends on genetic variation related to lactose intolerance as well as its interaction with dairy intake. On the contrary, studies reported that some intestinal probiotics could promote lactose metabolism and alleviated symptoms associated with lactose intolerance (16). Similarly, approximately 30% of the general population carries the HLA-DQ2/8 coeliac disease susceptibility gene; however, only 2–5% of these individuals go on to develop coeliac disease, suggesting that other environmental factors, including gut microbiota, contribute to the development of the disease (17). Caminero et al. (18) demonstrated that modified gluten peptides from *Pseudomonas aeruginosa* activated gluten-specific T cells in patients with Crohn’s disease. In contrast, *Lactobacillus* spp. from the duodenum of non-coeliac disease patients regulated the production of degraded gluten peptides by human proteases and *Pseudomonas aeruginosa* proteases, thereby reducing their immunogenicity (18). In addition, fecal culture and duodenal biopsies showed an increased abundance of Gram-negative bacteria, *Bacteroides*, *Clostridium*, and *E. coli* in patients with coeliac disease compared to healthy adults (19). The mechanisms through which gut microbiota influences the onset or progression of coeliac disease may involve activation of the innate immune system responses, modulation of epithelial barrier, or exacerbation of an alcohol-soluble protein-specific immune response (17). However, establishing a causal relationship between gut microbiota and lactose intolerance as well as coeliac disease may be limited due to various confounding factors such as environment, dietary habits and lifestyle.

Mendelian randomization (MR) offers an alternative approach for investigating causality in epidemiological studies by modifying exposure to associated genetic variation to assess causal outcomes, reducing confusion and potential bias in reverse causality (20, 21). In recent years, the causal relationship between gut microbiota with lactose intolerance as well as coeliac disease has remained unclear. Moreover, gut microbiota is susceptible to confounding factors, and observational studies restrict our ability to explore causality effectively. Hence, in this study, we employed MR analysis to investigate the relationship between genetic variation in the gut microbiota and lactose intolerance as well as coeliac disease.

## 2 Materials and methods

### 2.1 Study design

**Figure 1:**
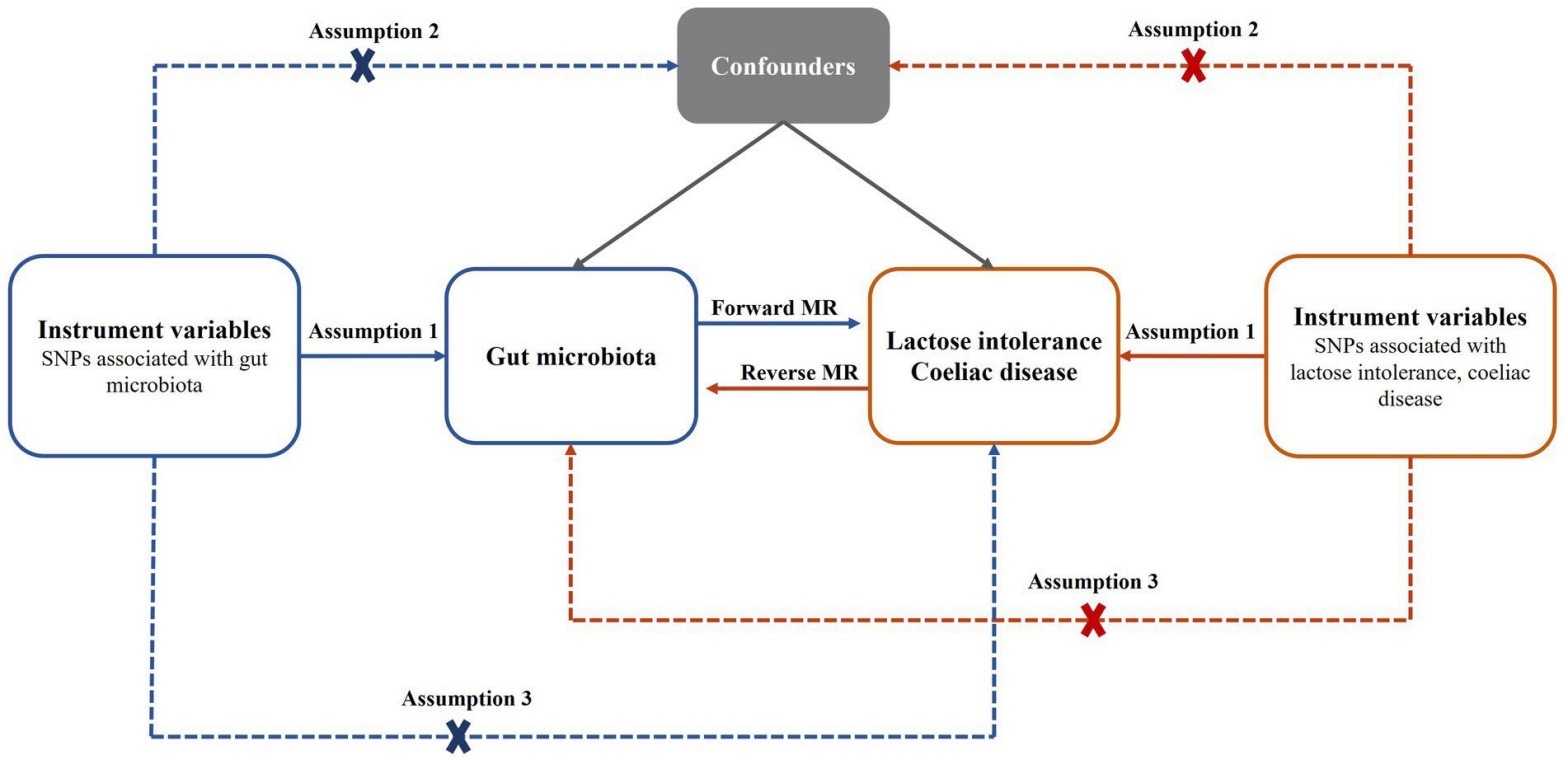
Bidirectional MR analysis process and three basic assumptions. SNPs, single nucleotide polymorphisms; MR, Mendelian randomization.

In this study, a bidirectional two-sample MR analysis was used to investigate the causal relationship between gut microbiota and lactose intolerance as well as coeliac disease. The study flow chart is depicted in Figure 1. Subsequently, reverse MR Analysis was conducted to explore whether lactose intolerance or coeliac disease exerted a causal effect on the identified microbiome, with lactose intolerance and coeliac disease being considered as exposures.

### 2.2 Data sources

Our study obtained single nucleotide polymorphisms (SNPs) associated with the gut microbiota as instrumental variables (IVs) from the genome-wide association study (GWAS) dataset of the International Consortium MiBioGen^1^. The consortium analyzed genotyping data and 16S rRNA gene sequencing profiles from 18,340 participants across 24 cohorts encompassing countries such as the United States, Canada, Israel, South Korea, Germany, Denmark, the Netherlands, Belgium, Sweden, Finland, and the United Kingdom (22). This study incorporated 211 taxa, which consisted of 9 phyla, 16 classes, 20 orders, 35 families, and 131 genera.

In our study, we focused on the trait “lactose intolerance.” GWAS data for lactose intolerance were sourced from the GWAS Catalog^2^ under study accession “GCST90044159.” The analysis encompassed a total of 456,348 individuals of European (U.K.) descent, comprising 150 cases of European ancestry and 456,198 controls of European ancestry (23). The phenotype “coeliac disease” was adopted in our research. GWAS data for coeliac disease were obtained from the FinnGen database (24), and the GWAS consisted of 3,690 cases (including 2,456 females and 1,234 males) and 361,055 controls. Our dataset was sourced from distinct databases to minimize the possibility of sample overlap.

### 2.3 Selection of IVs

Our choice of IVs conforms to the three basic assumptions of MR analysis as follows: (1) the relevance assumption: SNPs were directly associated with exposure; (2) the independence assumption: SNPs do not affect outcome through confounders; (3) the exclusion restriction assumption: SNPs affect outcomes only through exposure (25). To ensure a strong correlation between SNPs and exposure and to be able to obtain a sufficient number of IVs, we set a genome-wide statistical significance threshold of *p* < 1.0 × 10^−5^ to screen for SNPs associated with exposure. For the IVs used for reverse MR analysis, we set the genome-wide statistical significance threshold to *p* < 5.0 × 10^−8^, but did not filter a sufficient number of IVs for lactose intolerance, so we adjusted it to *p* < 1.0 × 10^−5^ for lactose intolerance. Then we performed a linkage disequilibrium (LD) analysis clumping SNPs to ensure independence among the selected SNPs. The chain imbalance threshold was set to *r*^2^ < 0.001, and the clumping window size was set to 10,000 kb, using a reference panel from the European 1,000 Genomes Projects. Next, we removed IVs with *F*-statistics less than 10 [*F* = (beta/se)2] to avoid bias caused by weak IVs (26). Proxies are not used for IVs that are not successfully harmonized.

To avoid the influence of confounding factors, we used the PhenoScanner online tool (PhenoScanner^3^) to query for traits associated with the selected SNPs and reviewed the literature to check whether these traits affect the outcome. The filtered SNPs should satisfy *p* > 1.0 × 10^−5^ in the outcome to ensure their independence for the outcome. Finally, we removed SNPs with palindromic structures.

### 2.4 MR analysis

We performed two-sample MR analyses to investigate the causal relationship between gut microbiota and lactose intolerance and coeliac disease. For the reverse MR analysis, we used two diseases as exposures and gut microbiota as outcomes. This approach allows us to assess whether there is a bidirectional causal link between gut microbiota and the two diseases. For the MR analysis, we used inverse variance weighted (IVW), MR Egger, Weighted median, Simple mode, and Weighted mode methods for evaluation. When SNPs perfectly adhered to the three assumptions of the MR study, the IVW method exhibited prior statistical power compared to all other methods, and therefore, we gave priority to the results obtained by the IVW method (27). A significance level of *p* < 0.05 was considered to determine whether potential causal effects were statistically significant.

### 2.5 Sensitivity analysis

Cochrane’s *Q* test was utilized to evaluate the presence of heterogeneity in the IVs, with significance considered when *p* < 0.05. The assessment of potential horizontal pleiotropy involved examining the intercept of the MR-Egger regression and MR-PRESSO, with horizontal pleiotropy deemed present when *p* < 0.05. Furthermore, we investigated whether a single IV was the driving factor behind the results through the “leave-one-out” method.

All statistical analyses were done using R software (version 4.3.2). MR and sensitivity analyses were done using the “TwoSampleMR” package (version 0.5.7) (28) and the “MRPRESSO” package (version 1.0) (29).

## 3 Results

### 3.1 IVs for analysis

Following the screening SNPs based on the locus-wide significance threshold of *p* < 1.0 × 10^−5^, we collected the data of 211 gut microbiota taxa from the MiBioGen official website. A total of 13,671 SNPs from 196 gut microbiota were used for the subsequent analyses after excluding SNP data from 15 unknown taxa. Then IVs with palindromic structures were eliminated, and the remaining SNPs were required to have *p* > 1.0 × 10^−5^ in the outcome data.

This process resulted in 2,106 SNPs related to lactose intolerance and 2,078 SNPs associated with coeliac disease from 196 gut microbiota taxa for subsequent MR analysis, with detailed information presented in Supplementary Tables S1, S2. Furthermore, the SNPs utilized for reverse MR analysis can be found in Supplementary Tables S3, S4.

### 3.2 Causal effect of gut microbiota on lactose intolerance and coeliac disease

**Figure 2:**
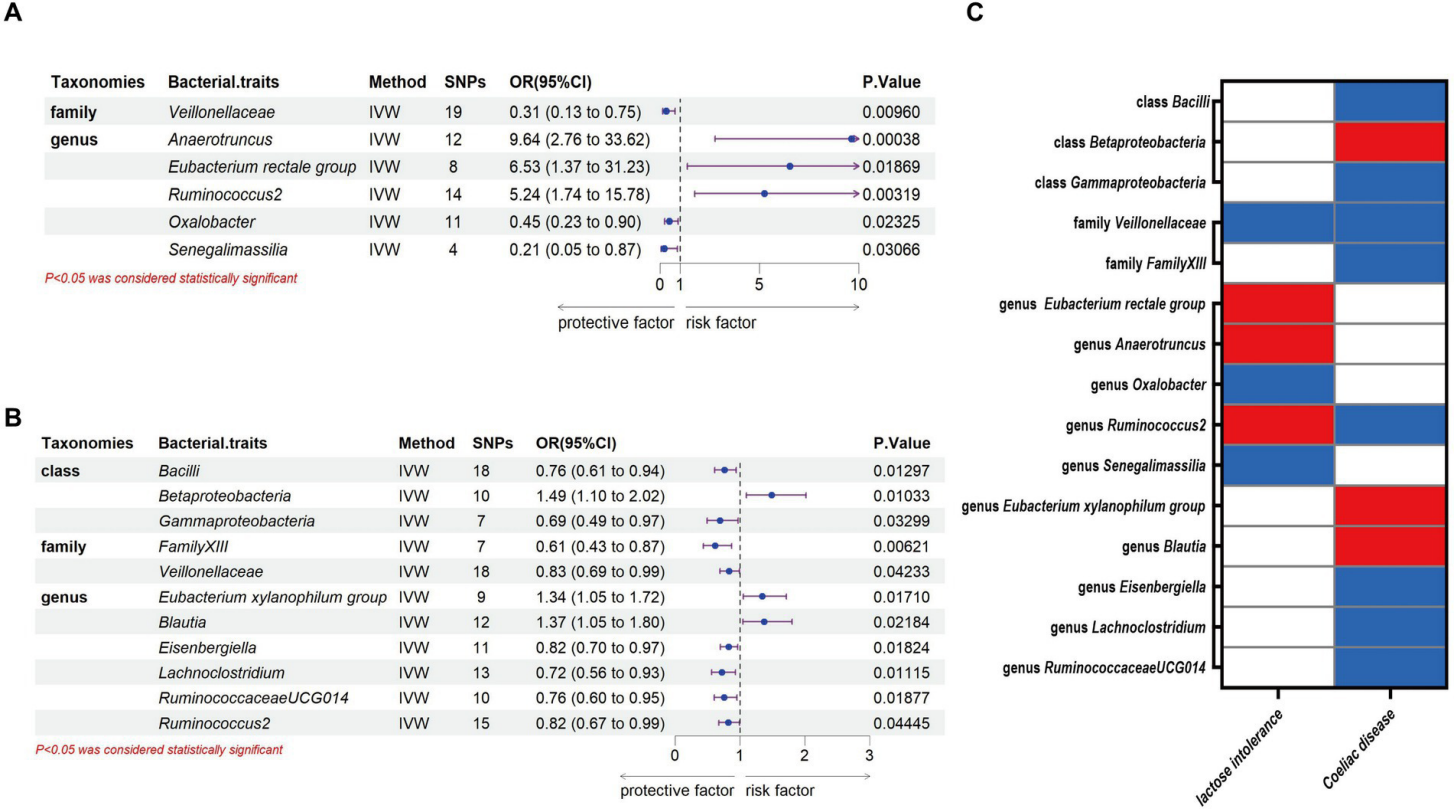
Causal association between gut microbiota and lactose intolerance and coeliac disease. **(A)** Causal association between gut microbiota and lactose intolerance. **(B)** Causal association between gut microbiota and coeliac disease. **(C)** Positive IVW-MR results was shown with heatmap with red indicating risk factors and blue indicating protective factors, respectively. IVW, inverse variance weighted; SNPs, single nucleotide polymorphisms; OR, odds ratio; CI, confidence interval.

Through the MR analysis, we discovered that 1 family and 5 genera had a causal relationship with lactose intolerance. Specifically, at the family level, the IVW analysis indicated a negative correlation between *Veillonellaceae* [odds ratio (OR) = 0.31, 95% confidence interval (95% CI), 0.13–0.75, *p* = 0.0096] and the occurrence of lactose intolerance. At the genus level, *Anaerotruncus*, (OR = 9.64, 95% CI, 2.76–33.62, *p* = 0.00038), *Eubacterium rectale group* (OR = 6.53, 95% CI, 1.37–31.23, *p* = 0.01869) and *Ruminococcus2* (OR = 5.24, 95% CI, 1.74–15.78, *p* = 0.00319) were positively correlated with lactose intolerance, while *Oxalobacter* (OR = 0.45, 95% CI, 0.23–0.90, *p* = 0.02325) and *Senegalimassilia* (OR = 0.21, 95% CI, 0.05–0.87, *p* = 0.03066) were negatively correlated with lactose intolerance (Figure 2A).

Meanwhile, we identified 3 classes, 2 families and 6 genera that exhibited a causal relationship with coeliac disease. Specifically, at the class level, *Bacilli* (OR = 0.76, 95% CI, 0.61–0.94, *p* = 0.01297) and *Gammaproteobacteria* (OR = 0.69, 95% CI, 0.49–0.97, *p* = 0.03299) showed a negative association to the development of coeliac disease, while there was a positive association of *Betaproteobacteria* (OR = 1.49, 95% CI, 1.10–2.02, *p* = 0.01033). At the family level, we identified both *FamilyXIII* (OR = 0.61, 95% CI, 0.43–0.87, *p* = 0.00621) and *Veillonellaceae* (OR = 0.83, 95% CI, 0.69–0.99, *p* = 0.04233) as having a negative association with the development of coeliac disease. At the genus level, *Eubacterium xylanophilum group* (OR = 1.34, 95% CI, 1.05–1.72, *p* = 0.0171) and *Blautia* (OR = 1.37, 95% CI, 1.05–1.80, *p* = 0.01869) were positively correlated with coeliac disease, while *Eisenbergiella* (OR = 0.82, 95% CI, 0.70–0.97, *p* = 0.01824), *Lachnoclostridium* (OR = 0.72, 95% CI, 0.56–0.93, *p* = 0.01115), *RuminococcaceaeUCG014* (OR = 0.76, 95% CI, 0.60–0.95, *p* = 0.01877) and *Ruminococcus2* (OR = 0.82, 95% CI, 0.67–0.99, *p* = 0.04445) were negatively correlated with coeliac disease (Figure 2B).

**Figure 3:**
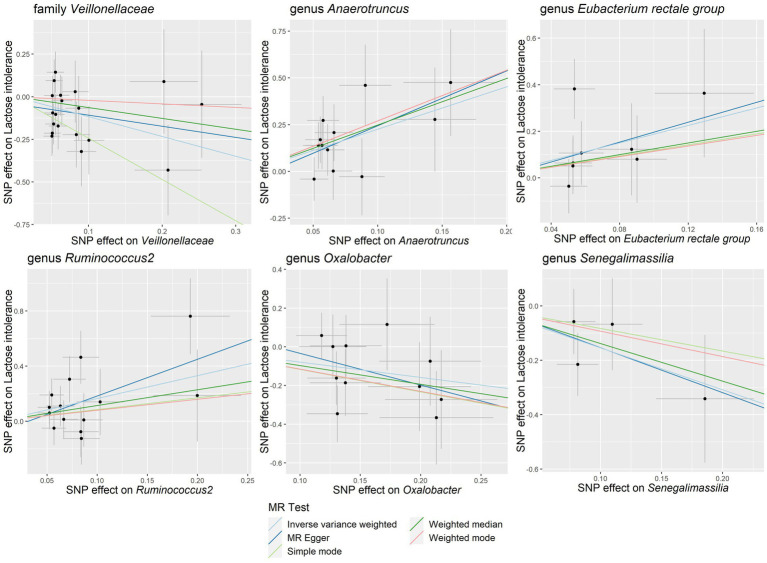
Scatter plots of the causal association between gut microbiota and lactose intolerance. SNP, single nucleotide polymorphism; MR, Mendelian randomization.

**Figure 4:**
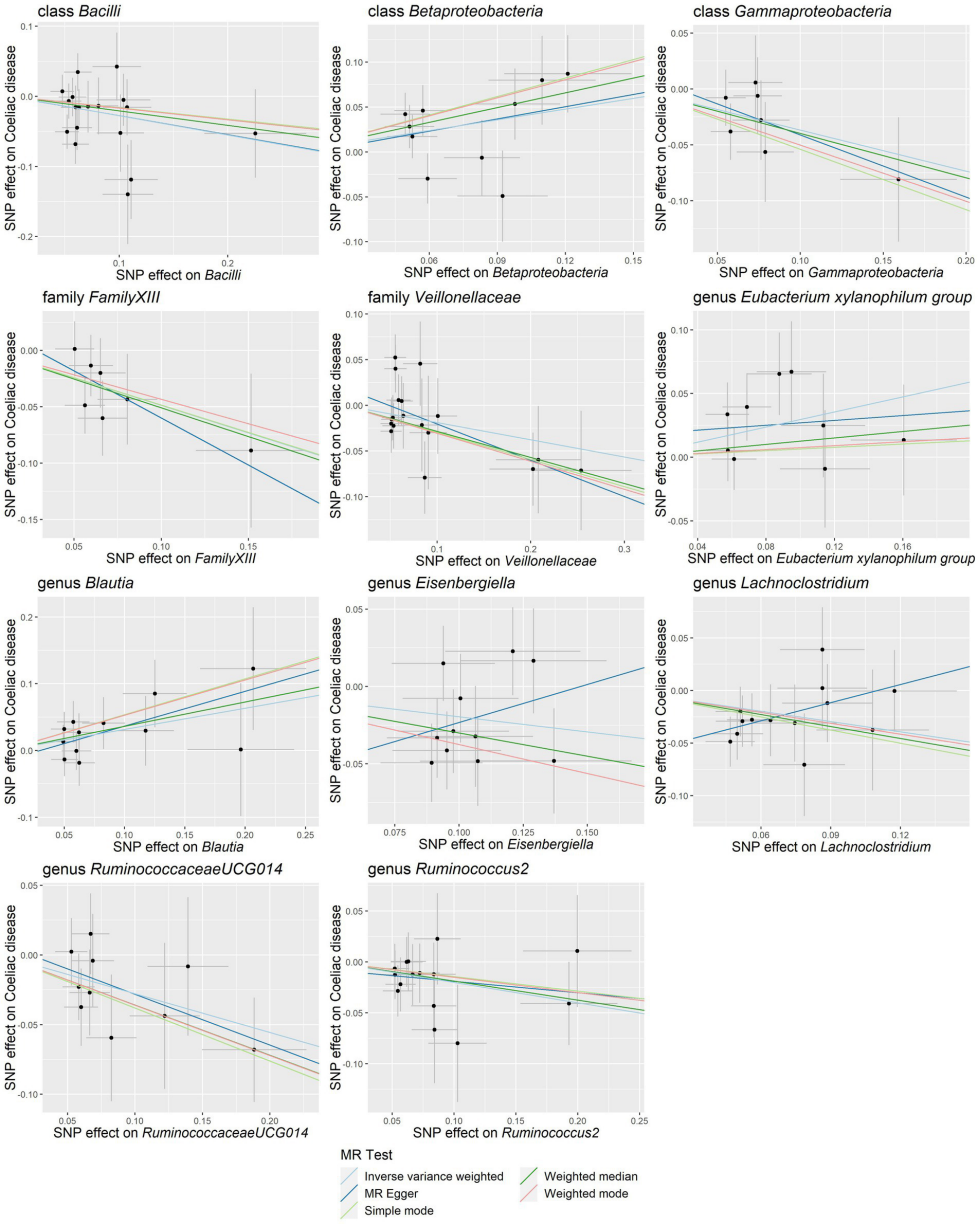
Scatter plots of the causal association between gut microbiota and coeliac disease. SNP, single nucleotide polymorphism; MR, Mendelian randomization.

Our study notably unveiled a causal relationship between the family *Veillonellaceae* and genus *Ruminococcus2* in relation to both lactose intolerance and coeliac disease. *Veillonellaceae* emerged as a common protective factor for both conditions, whereas *Ruminococcus2* was identified as a risk factor for lactose intolerance but a protective factor for coeliac disease (Figure 2C). The scatter plots of MR analysis for lactose intolerance and coeliac disease can be found in Figures 3, 4. More comprehensive details of the MR results are available in Supplementary Tables S5, S6. Additionally, an alternative representation of the effects of SNPs can be observed in Supplementary Figures S1, S2, which features a forest plot.

### 3.3 Sensitivity analysis

**Figure 5:**
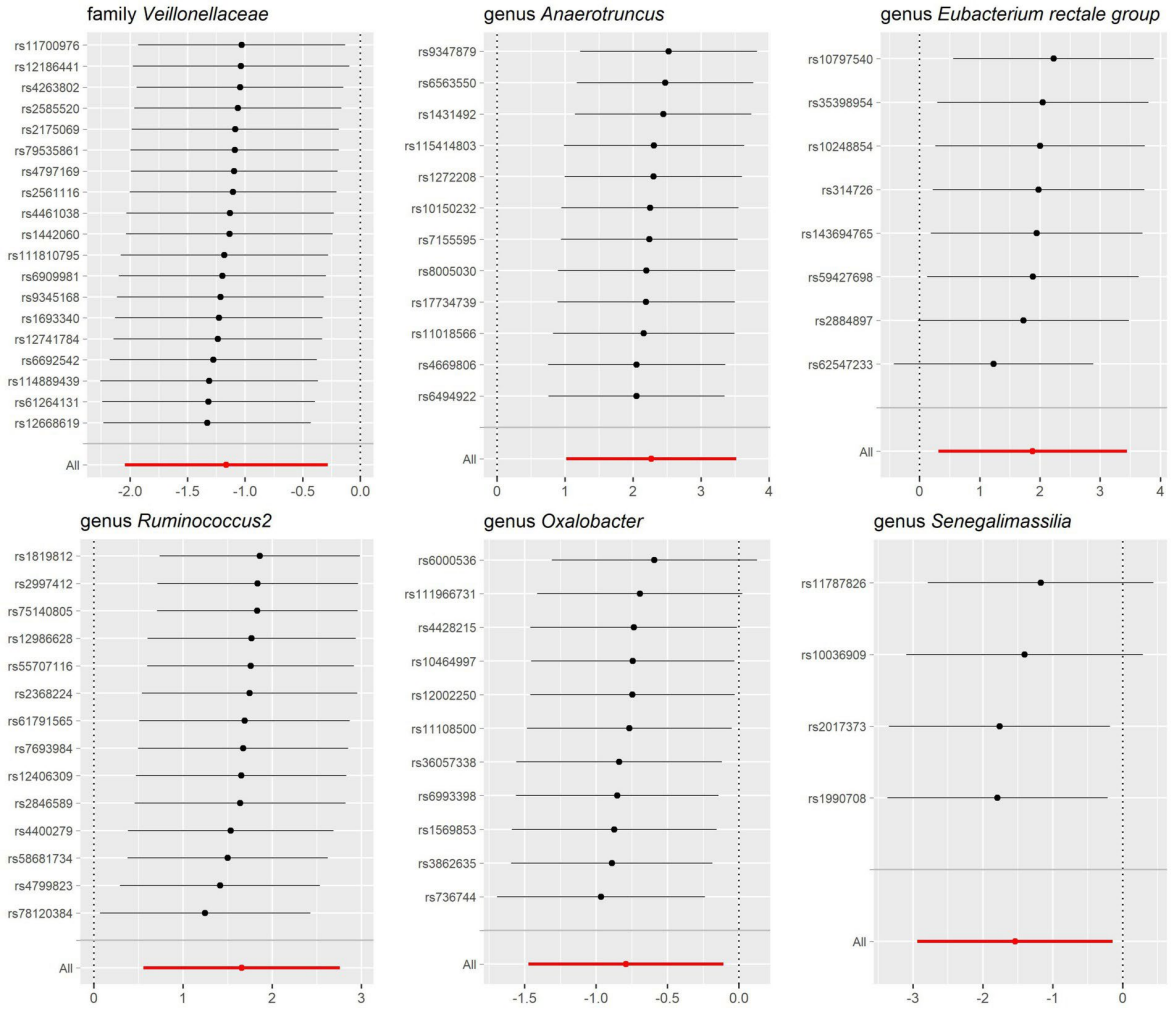
Leave-one-out sensitivity analysis for gut microbiota on lactose intolerance. Error bars indicate 95% confidence interval.

**Figure 6:**
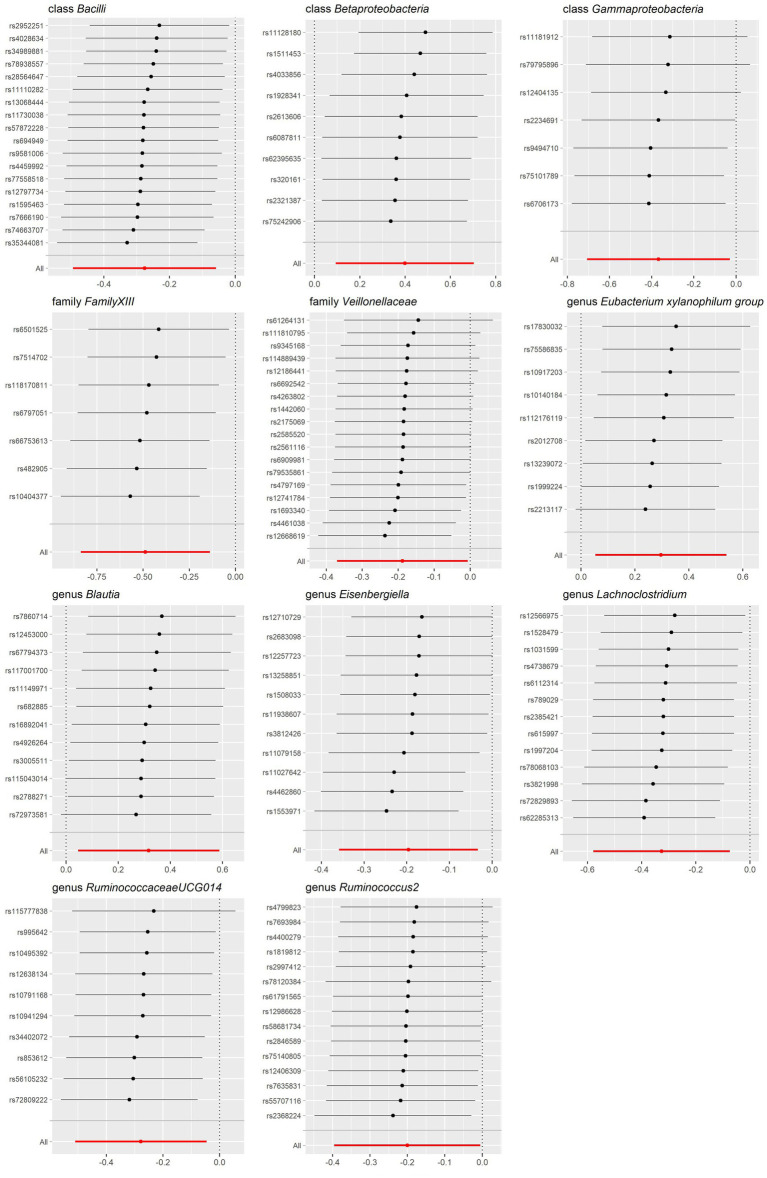
Leave-one-out sensitivity analysis for gut microbiota on coeliac disease. Error bars indicate 95% confidence interval.

The sensitivity analysis conducted in our study aimed to ensure the reliability and robustness of the results obtained. In Cochrane’s *Q* test, *p-*values were all >0.05, indicating that heterogeneity bias did not significantly influence the outcomes (Tables 1, 2). Moreover, the findings from both the MR-Egger intercept and MR-PRESSO Global Test, showing *p*-values >0.05, suggest the absence of horizontal pleiotropy, affirming that the IVs did not impact the risk of lactose intolerance or coeliac disease through pathways unrelated to gut microbiota (Tables 1, 2). For a comprehensive view of the sensitivity analyses conducted on all taxa and their association with lactose intolerance and coeliac disease, please refer to Supplementary Tables S7–S12. The leave-one-out analysis indicated that no individual IV significantly influenced the overall estimates for either condition, as depicted in Figures 5, 6.

**Table 1:** Sensitivity analysis between gut microbiota and lactose intolerance.

Taxonomies	Bacterial traits	SNPs	Heterogeneity test			Horizontal pleiotropy test		
			Cochrane’s *Q*			MR-Egger Intercept		MR-PRESSO Global Test
			Method	*Q*	*P*	Intercept	*P*	*P*
Family	*Veillonellaceae*	19	MR-Egger	15.223	0.579	−0.043	0.569	0.664
			IVW	15.560	0.623			
Genus	*Anaerotruncus*	12	MR-Egger	6.922	0.733	−0.048	0.734	0.832
			IVW	7.044	0.795			
	*Eubacterium rectale group*	8	MR-Egger	6.690	0.350	−0.017	0.930	0.551
			IVW	6.699	0.461			
	*Ruminococcus2*	14	MR-Egger	12.768	0.386	−0.082	0.485	0.428
			IVW	13.321	0.423			
	*Oxalobacter*	11	MR-Egger	7.471	0.588	0.130	0.600	0.652
			IVW	7.766	0.652			
	*Senegalimassilia*	4	MR-Egger	1.288	0.525	0.012	0.964	0.760
			IVW	1.291	0.731			

SNPs, single nucleotide polymorphisms; IVW, inverse variance weighted.

**Table 2:** Sensitivity analysis between gut microbiota and coeliac disease.

Taxonomies	Bacterial traits	SNPs	Heterogeneity test			Horizontal pleiotropy test		
			Cochrane’s *Q*			MR-Egger Intercept		MR-PRESSO Global Test
			Method	*Q*	*P*	Intercept	*P*	*P*
Class	*Bacilli*	18	MR-Egger	18.817	0.278	0.000	0.989	0.389
			IVW	18.817	0.339			
	*Betaproteobacteria*	10	MR Egger	10.501	0.232	−0.004	0.915	0.363
			IVW	10.517	0.310			
	*Gammaproteobacteria*	7	MR-Egger	2.078	0.838	0.015	0.736	0.905
			IVW	2.205	0.900			
Family	*FamilyXIII*	7	MR-Egger	2.798	0.731	0.023	0.613	0.824
			IVW	3.089	0.798			
	*Veillonellaceae*	18	MR-Egger	15.382	0.497	0.019	0.213	0.504
			IVW	17.064	0.450			
Genus	*Eubacterium xylanophilum group*	9	MR-Egger	5.513	0.598	0.017	0.584	0.692
			IVW	5.842	0.665			
	*Blautia*	12	MR-Egger	5.579	0.849	−0.016	0.546	0.881
			IVW	5.969	0.875			
	*Eisenbergiella*	11	MR-Egger	9.164	0.422	−0.073	0.281	0.392
			IVW	10.504	0.397			
	*Lachnoclostridium*	13	MR-Egger	4.250	0.962	−0.063	0.054	0.708
			IVW	8.898	0.712			
	*RuminococcaceaeUCG014*	10	MR-Egger	3.882	0.868	0.008	0.735	0.935
			IVW	4.005	0.911			
	*Ruminococcus2*	15	MR-Egger	5.354	0.967	−0.008	0.693	0.982
			IVW	5.517	0.977			

SNPs, single nucleotide polymorphisms; IVW, inverse variance weighted.

### 3.4 Reverse MR analysis

In the reverse MR analysis focusing on the gut microbiota composition identified in the forward MR analysis, no evidence of reverse causality between lactose intolerance and coeliac disease was found across various analytical methods, as outlined in Supplementary Tables S13, S14. Subsequent Cochrane’s *Q* test did not reveal the presence of heterogeneity, and MR-Egger regression intercept analysis and MR-PRESSO Global Test did not reveal the presence of horizontal pleiotropy (Supplementary Tables S15–S20), thus supporting the robustness of the findings. In summary, the reverse MR analysis confirmed the absence of causal relationships between the gut microbiota taxa and the studied diseases.

## 4 Discussion

There are approximately 40 trillion bacteria in the human body, approximately 99% of which are contained in the human colon. Gut microbiota homeostasis is essential for nutrient absorption, and the imbalance of gut microbiota may lead to impaired nutrient absorption. Evidence has shown that gut microbiota play a pivotal role in the progression of lactose intolerance and coeliac disease (12, 17). In this study, MR analysis was conducted using GWAS data from the International Consortium MiBioGen database, the GWAS Catalog, and the FinnGen database to investigate the causal relationship between the gut microbiota and lactose intolerance as well as coeliac disease. Overall, we identified 6 microorganism taxa for lactose intolerance and 11 microorganism taxa for coeliac disease with either positive or negative effect. This study establishes a potential causal link between the composition of the gut microbiota and the development of lactose intolerance and coeliac disease, offering a fresh perspective on the pathogenesis of these conditions and pointing toward novel therapeutic targets.

For lactose intolerance, we found that family *Veillonellaceae*, genus *Oxalobacter* and *Senegalimassilia* were protective factors for lactose intolerance, while genus *Anaerotruncus*, *Eubacterium rectale group* and *Ruminococcus2* were identified as risk factors. Family *Veillonellaceae*, a group of Gram-negative bacteria in Firmicutes, was known to ferment lactic acid and produced acetate, propionate, and carbon monoxide (30). Firrman et al. (31) harvested fecal samples from 18 donors and performed anaerobic culture in the presence or absence of lactose, and they found that the abundance of *Veillonellaceae* increased as lactose intake increased indicating that *Veillonellaceae* was closely related to lactose utilization. Our study identified *Veillonellaceae* to be a protective factor for lactose intolerance. Therefore, these suggest that *Veillonellaceae* involved lactose utilization disorder plays a part in the mechanism of lactose intolerance. Genus *Oxalobacter*, a protective factor for lactose intolerance in our study, was reported to degrade intestinal oxalate and prevent hyperoxaluria (30). Kumar et al. (32) found that the abundance of *Oxalobacter* was reduced in the feces of patients with inflammatory bowel disease (IBD). These suggested that *Oxalobacter* might protect against some gastrointestinal diseases (32). Regarding *Senegalimassilia*, Jiang et al. found that genus *Senegalimassilia* exhibited a protective effect against pancreatic cancer (33). Adamberg et al. (34) observed *Senegalimassilia* exhibited enhanced growth in fecal samples of normal-weight children upon supplementation with sugar substrates, contrasting with the findings in overweight children. In addition, the genome of *Senegalimassilia* encodes proteins involved in glycolysis (phosphofructokinase) and sugar transport (sugar ABC transporter proteins), suggesting a potential for sugar utilization (34). But the direct role of *Oxalobacter* and *Senegalimassilia* in lactose utilization or lactose intolerance has not been reported. Genus *Anaerotruncus*, has been reported to be positively associated with the risk of several diseases, such as nervous system diseases, obesity and liver cancer (35–37). *Anaerotruncus*, a conditionally pathogenic bacterium, was shown to be increased in the feces of mice on a high sucrose diet and was linked to sugar metabolism dysregulation (36). Ye et al. (38) found gut microbes and aging, and identified genus *Eubacterium rectale group* to be a frailty-related taxon. Meanwhile, a comparative study conducted by Di Stefano et al. (39) showed that the prevalence of lactose malabsorption tended to increase with age. These results indicate a possible age-mediated link between *Eubacterium rectale group* and lactose intolerance (38, 39). But there is no direct evidence to prove that *Anaerotruncus* and *Eubacterium rectale group* are associated with the development of lactose intolerance.

For coeliac disease, microorganism taxa including class *Bacilli* and *Gammaproteobacteria*, family *FamilyXIII* and *Veillonellaceae*, genus *Eisenbergiella*, *Lachnoclostridium*, *RuminococcaceaeUCG014* and *Ruminococcus2* displayed significant protective effects. Conversely, class *Betaproteobacteria*, genus *Eubacterium xylanophilum group*, and genus *Blautia* were associated with increased risk of coeliac disease. Class *Bacilli* was found to be a protective factor for coeliac disease in our study, which could be supported by previous research. De Angelis et al. (40) identified that *Bacilli* could improve gluten digestion in gluten-sensitive patients by hydrolyzing immunogenic peptides during gastrointestinal digestion. Khan et al. (41) demonstrated that *Bacillus subtilis* LZU-GM alleviated the adverse effects of gluten-added foods in mice and balanced the gut microbiota in mice. Moreover, this study showed reduced expression of IFN-γ, TNF-α, IL-10, and COX-2 in the lamina propria of mice treated with *Bacillus subtilis* (41). The results above suggested that *Bacilli*, particularly *Bacillus subtilis*, might have beneficial effects in improving gluten digestion and balancing gut microbiota, thus potentially alleviating the adverse effects associated with gluten consumption, with the possible mechanism in mitigating inflammation at the gastrointestinal level. In the gut microbiota of patients with active coeliac disease, there was an increase in the abundance of *Betaproteobacteria*, while the abundance of *Gammaproteobacteria* decreased (42). At the family level, *FamilyXIII* and *Veillonellaceae* are protective against coeliac disease. There are few studies on *FamilyXIII*, but one study showed that *FamilyXIII UCG001* under this family was negatively correlated with serum levels of TNF-α (43). Therefore, we suggest that subpopulations under *FamilyXIII* may be involved in the regulation of inflammatory responses in humans. Research by Li et al. (44) demonstrated that *Veillonellaceae* could reduce intestinal inflammation by downregulating pro-inflammatory molecules such as IL-6, IL-1β, iNOS, and IFN-γ as well as oxidative stress markers like MDA and MPO. These suggest that *Veillonellaceae* may play a protective role in certain autoimmune diseases. Bonder et al. (45) investigated the gut microbiome of 21 healthy volunteers following a gluten-free diet and found a significant reduction in *Veillonellaceae* during the intervention. The pathogenic nature of the genus *Eubacterium xylanophilum* group, as indicated by its potential to inhibit the growth of SCFA-producing bacteria and its associations with metabolic disorders and colon cancer, raised concerns regarding its role in coeliac disease (46). Butyric acid has shown to be an important SCFA associated with various diseases. Butyric acid has a positive metabolic effect on enterocytes and is beneficial to intestinal barrier function. Moreover, butyric acid has been demonstrated to alleviate inflammation of the gastrointestinal mucosa (47). Therefore, we speculated that the pathogenic effect of *Eubacterium xylanophilum group* in coeliac disease might be mediated by SCFA. At the same time, *Rabdosia serra*, an herbal tea ingredient, was found to alleviate dextran sulfate sodium (DSS) salt-induced colitis in mice by decreasing the abundance of *Eubacterium xylanophilum group* and other pathogenic bacteria, regulating the composition of intestinal microbiota (48). Genus *Blautia*, a mucin degrader, was observed to be increased in IBD and primary sclerosing cholangitis (PSC) compared to healthy controls (49). Garay et al. (50) found that increased *Blautia* abundance was associated with the risk of developing Crohn’s disease, while reduced level of *Blautia* was reported in the feces of patients with coeliac disease, but the further mechanism has not been elucidated (51). This phenomenon may result from host feedback regulation of pathogenic bacterial abundance. Gryaznova et al. (52) observed a significant reduction in the genus *Eisenbergiella* among patients with ulcerative colitis. *Eisenbergiella* is known to play a crucial role in producing butyrate, which is the primary energy source for intestinal epithelial cells (53, 54). Butyrate has been shown to have anti-inflammatory properties in the gastrointestinal mucosa, potentially contributing to its protective effects against coeliac disease. *Lachnoclostridium* is a crucial component of the human gut microbiome, playing a role in maintaining homeostasis and exhibiting anti-inflammatory properties. Its metabolites, such as butyrate, alleviate colitis in mice by altering the distribution of intraepithelial lymphocytes, thereby promoting intestinal health and inducing the differentiation and expansion of regulatory T cells (55–57). Clinical studies had shown that *Lachnoclostridium* abundance is significantly lower in patients with IBD compared to healthy individuals (58). According to Stene et al. (59), recurrent rotavirus infection was a predictor of higher risk of coeliac disease autoimmunity. *Lachnoclostridium* abundance was reduced in rotavirus-infected children with diarrhea. This suggests that viral infections early in life may affect immune development by affecting the gut microbiota, thereby causing coeliac disease (60). However, direct evidence supporting this relationship is currently lacking in the existing studies. Limited information is available on the genus *RuminococcaceaeUCG014*. One study stated that glycine could ameliorate LPS-induced intestinal injury in mice with an increased abundance of *RuminococcaceaeUCG014*, implying its possible protective effect of *RuminococcaceaeUCG014* against intestinal injury (61). Yu et al. (62) demonstrated a negative correlation between the abundance of *RuminococcaceaeUCG014* and the expression of pro-inflammatory cytokines (IL-1a, IL-6, IL-12a, IL-12b, IL-17a), suggesting a potential role in reducing intestinal inflammation. Regarding genus *Ruminococcus2*, Wang et al. (63) found that its depletion was one of the alterations in gut microbiology in autoimmune diseases. In an observational study conducted by Li et al. (64), the abundance of *Ruminococcus2* was higher in patients with lower levels of T cell and B cell. However, its effects on coeliac disease have been rarely reported.

Our study elucidates the independent effects of different bacteria on lactose intolerance and coeliac disease. However, it is worth emphasizing that the human digestive system is complex and the role of the microorganisms (such as archaea, viruses, and fungi) should also be taken into account. Archaea has a strong pro-inflammatory effect and a strong correlation with TNF-α, which may be a risk factor for coeliac disease (65, 66). Viruses, as mentioned earlier, may influence disease progression by altering the composition of the gut microbiota. As for fungi, Harnett et al. detected *Candida* sp. in 33% of fecal specimens from coeliac disease patients compared to 0% of controls, suggesting that *Candida* may act as a trigger for autoimmune responses in genetically susceptible people (67). There is a similarity between the hyphal wall protein 1 of *Candida albicans* and the coeliac disease-associated gliadin T-cell epitope (68, 69). This hypothesis was supported by higher serum levels of anti-hyphal wall protein 1, anti-gliadin and anti-tissue transglutaminase 2 antibodies in coeliac disease patients than in healthy controls (70). Diet is another important factor influencing gut microbial composition. Dairy consumption was positively correlated with *Saccharomyces* abundance, and carbohydrate intake was positively correlated with *Candida* and *Methanobrevibacter* abundance (71, 72). Lactose and gluten, while acting as pathogenic factors, and their insufficient intake may also affect the composition of gut microorganisms and their interactions, further influencing the disease. The interactions between different gut microbiomes should be elucidated in future studies of lactose intolerance and coeliac disease.

This study has several strengths. The utilization of MR analysis enables the identification of causal relationships between gut microbiota and lactose intolerance as well as coeliac disease, thereby enhancing the capacity to infertility by eliminating the impact of confounding variables and the potential for reverse causation. The genetic variation data pertaining to gut microbiota lactose intolerance and coeliac disease were derived from the largest GWAS meta-analysis of published papers, ensuring the reliability of the IVs used for MR analysis. Moreover, the GWAS data were obtained from diverse databases, including MiBioGen, GWAS Catalog, FinnGen database, thereby mitigating the bias resulting from sample overlap between the exposure and the outcome. During the IV selection process, we augmented the *F*-statistic to ensure the strength of SNPs and filtered out confounding SNPs using the PhenoScanner online tool. Analyses were subjected to Cochrane’s *Q*-test to assess for heter and tested for horizontal pleiotropy through MR-Egger regression and MR-PRESSO. Nevertheless, it is important to acknowledge that this study has certain limitations. Ethnic disparities in the prevalence of lactose intolerance and coeliac disease, and we were unable to narrow down our analysis to specific ethnic groups due to limitations in the available GWAS data. Despite we tried to select as much sample data as possible, the relatively small sample size of case data may still impose certain limitations when performing MR analysis. In addition, since our data samples primarily originate from European populations, caution should be exercised when generalizing their relevance to other populations.

## 5 Conclusion

In conclusion, through MR analysis, we have identified specific taxa of gut microbiota associated with lactose intolerance and coeliac disease. For lactose intolerance, the family *Veillonellaceae*, genus *Oxalobacter* and *Senegalimassilia* were found to be protective factors, while the genus *Anaerotruncus*, *Eubacterium rectale group* and *Ruminococcus2* were identified as risk factors. In the case of coeliac disease, the class *Bacilli* and *Gammaproteobacteria*, family *FamilyXIII* and *Veillonellaceae*, as well as the genera *Eisenbergiella*, *Lachnoclostridium*, *RuminococcaceaeUCG014* and *Ruminococcus2,* were considered protective factors. Conversely, the class *Betaproteobacteria*, genus *Eubacterium xylanophilum group* and *Blautia* were designated as risk factors for coeliac disease. Additionally, reverse MR analysis indicated that lactose intolerance or coeliac disease did not influence the abundance of the aforementioned bacteria. This study highlights the potential role of gut microbiota in the pathogenesis and treatment of lactose intolerance and coeliac disease, suggesting new avenues for research in this area.

While MR analysis established statistical causality to some extent, providing valuable evidence for the relationship between exposure and outcome, it is essential to note that definitive causality cannot be conclusively proven. The precise protective or risk effects of the identified gut microbiota on lactose intolerance and coeliac disease warrant further investigation through cohort studies or fundamental medical research in the future.

## Data availability statement

The original contributions presented in the study are included in the article/Supplementary material, further inquiries can be directed to the corresponding author.

## Author contributions

ZH: Conceptualization, Formal analysis, Investigation, Methodology, Software, Writing – original draft. YR: Conceptualization, Writing – review & editing. JLi: Writing – original draft. XZ: Validation, Writing – review & editing. HY: Validation, Writing – review & editing. JLiu: Methodology, Writing – review & editing. SD: Formal analysis, Writing – review & editing. HJ: Resources, Writing – review & editing. ZY: Data curation, Writing – review & editing. YL: Methodology, Writing – review & editing. LG: Data curation, Writing – review & editing. SZ: Writing – review & editing. SB: Writing – review & editing, Supervision. WY: Writing – review & editing. BW: Writing – review & editing. LZ: Writing – review & editing.

## Footnotes

^1^ https://www.mibiogen.org

^2^ https://www.ebi.ac.uk/gwas/

^3^ https://www.cam.ac.uk
