# Correction: Association of gut microbiota with lactose intolerance and coeliac disease: a two-sample Mendelian randomization study

**DOI:** 10.3389/fnut.2026.1850609

**Published:** 2026-04-28

**Authors:** Zongze Han, Ying Ran, Jiwen Li, Xue Zhang, Hui Yang, Jiangpeng Liu, Shijing Dong, Hao Jia, Zhen Yang, Yanni Li, Liping Guo, Simin Zhou, Suriguge Bao, Wei Yuan, Bangmao Wang, Lu Zhou

**Affiliations:** Department of Gastroenterology and Hepatology, General Hospital, Tianjin Medical University, Tianjin, China

**Keywords:** lactose intolerance, coeliac disease, gut microbiota, Mendelian randomization, genome-wide association study

An incorrect number was provided for the Natural Science Foundation of Tianjin [grant number 21JCZDJC00880]. The correct number is 23JCQNJC01300.

The original version of this article has been updated.

